# The utility of 1.5 tesla MR-guided adaptive stereotactic body radiotherapy for recurrent ovarian tumor – Case reports and review of the literature

**DOI:** 10.1016/j.ijscr.2022.107696

**Published:** 2022-09-22

**Authors:** Guler Yavas, Ulku Esra Kuscu, Ali Ayhan, Cagdas Yavas, Cem Onal

**Affiliations:** a Baskent University, School of Medicine, Department of Radiation Oncology, Turkey; b Baskent University, School of Medicine, Department of Obstetrics and Gynecology, Division of Gynecologic Oncology, Turkey; c Baskent University Faculty of Medicine, Adana Dr. Turgut Noyan Research and Treatment Center, Department of Radiation Oncology, Adana, Turkey

**Keywords:** Ovarian cancer, Oligometastasis, Radiotherapy, MR-guided radiotherapy, Stereotactic body radiotherapy

## Abstract

**Introduction:**

Although epithelioid ovarian cancer (EOC) is a radiosensitive tumor and radiotherapy (RT) played a significant role in adjuvant treatment management in the past, the role of RT has evolved with the advent of platinum-based chemotherapy regimens. Nonetheless, modern RT techniques may be useful in certain patients particularly those with recurrent disease.

**Presentation of case:**

After surgery and chemotherapy, two patients, aged 57 and 70, presented with recurrent lesions in the parailiac region. The recurrent lesions were treated with high field 1.5-Tesla MR-Linac treatment in 5 fractions at a dose of 30 Gy. The patients tolerated the treatment well and were disease free after 12 and 20 months of magnetic resonance guided radiotherapy (MRgRT), respectively.

**Discussion:**

MRgRT is a novel and rapidly evolving technology that allows for the highly precise treatment of even mobile targets through direct visualization of the tumor. The majority of patients with EOC frequently present with abdominal-pelvic recurrences. It has been demonstrated that EOC requires high radiation doses for curative treatment. MR-Linac enables monitoring of organ motion during treatment, which is necessary for delivering higher doses to target volumes while sparing surrounding organs.

**Conclusion:**

To reduce radiation doses to nearby normal tissues, MRgRT allows for the delivery of hypofractionated RT with tight safety margins. Regardless of initial resistance or gradual development of intolerance to standard chemotherapy regimens, the role of RT in patients with persistent or recurrent EOC should be reconsidered.

## Highlights

• Abdominal-pelvic region or other distant recurrence is common in ovarian cancer patients.
• The role of radiotherapy is debatable in ovarian cancer patients due to toxicity.
• Magnetic resonance guided RT allows direct target visualization during treatment delivery.
• We present two cases of recurrent ovarian cancer treated with adaptive MRgRT using 1.5 T-MR linac.
• MRgRT may be an alternative treatment option in patients with persistent or recurrent ovarian cancer.

## 1 Introduction

Ovarian cancer (OC) is one of the most prevalent cancers in women, accounting for 3 % of all cancer and 5 % of cancer-related deaths in the female population [1]. Epithelial ovarian cancer (EOC) is the most prevalent form of ovarian cancer (OC), and high-grade serous tumors account for approximately 70 % of EOS [2].

Despite significant advances in surgery and chemotherapy (ChT) over the past three decades, overall survival (OS) for patients with OC has not changed significantly. Although 60 % to 80 % of patients with advanced EOC respond initially to first-line ChT, approximately 70 % of patients experience recurrence in the abdominal-pelvic region or other distant sites [3], [4]. Significant advances have been made in the last decade in both systemic and surgical treatment of EOC. Nonetheless, the 5-year survival rate for FIGO stage III disease remains between 20 % and 25 %, and the median time to recurrence is less than two years [5]. In order to improve the oncological outcome of EOC patients, novel treatment strategies are required.

The EOC is accepted as a radiosensitive tumor, and radiotherapy (RT) played a significant role in adjuvant treatment management in the past. However, the function of RT has changed over time as platinum-based ChT regimens have become more prevalent. Modern irradiation techniques, on the other hand, may still play a role in certain patients with recurrent disease, particularly in cases of limited local recurrences and oligometastatic/oligo-recurrent disease. Particularly, the introduction of magnetic resonance guided RT (MRgRT) permits direct visualization of the target during treatment delivery, allowing for higher-dose administration with less damage to healthy tissue near the tumor [6]. We present two recurrent EOS cases that were treated with high field 1.5-Tesla (T) MR-linac using stereotactic MR-guided online adaptive radiation therapy (SMART).

The work was reported in accordance with SCARE criteria and the revised SCARE guidelines for 2020 [7].

## 2 Case presentations

### 2.1 Case 1

**Fig. 1:**
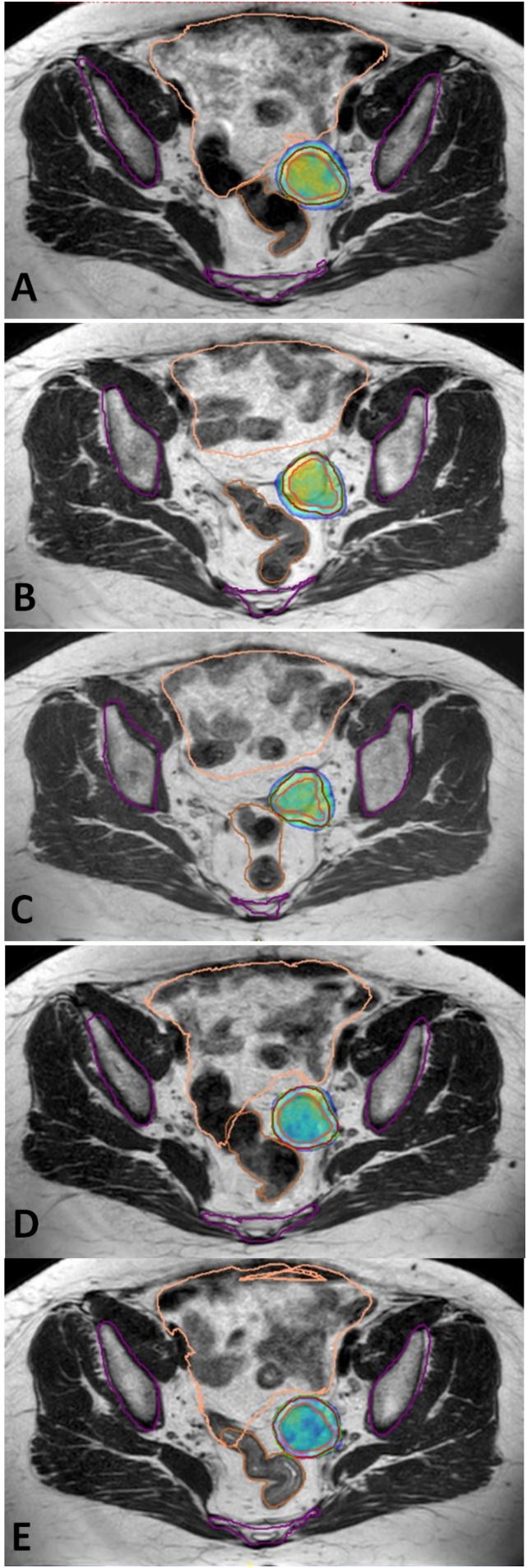
Recurrent lesion located at left parailiac area of Case 1 treated with MR-guided stereotactic radiotherapy using online adaptive radiation therapy delivered 30 Gy to recurrent lesion in 5 fractions. 95 % isodose volumes (green area) for each fraction of the adapt-to-shape adaptive plan (A–E). Gross tumor volume (pink line), planning target volume (red line), organs at risk are bones (purple line), rectum (orange line), and bowel bag (beige line). (For interpretation of the references to colour in this figure legend, the reader is referred to the web version of this article.)

**Fig. 2:**
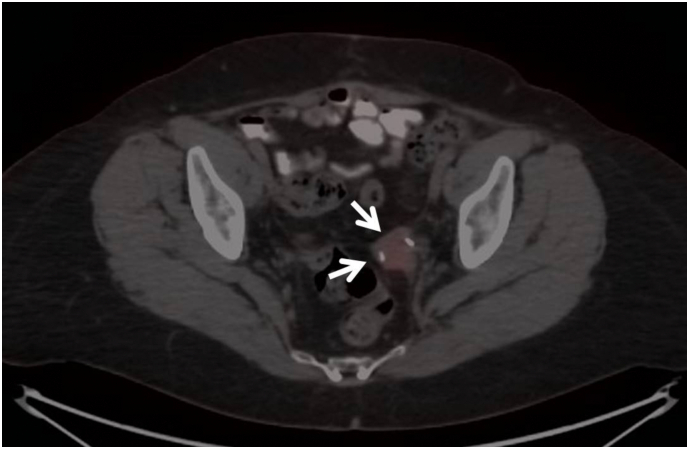
Complete metabolic response in lesion (arrow) demonstrated in positron emission tomography taken 6 months after completion of MR-guided stereotactic radiotherapy.

In 2013, a 57-year-old female with a history of EOC underwent total abdominal hysterectomy and a right salpingo-oophorectomy. She was admitted to the hospital in June 2016 with abdominal distension, pain, and a slight feeling of fullness after meals. She had left salpingo-oophorectomy, pelvic and para-aortic lymph node dissection, and appendectomy after being diagnosed with left-sided ovarian cancer. Histopathological findings revealed a stage II epithelial ovarian cancer with a high-grade serous subtype. Six cycles of chemotherapy with paclitaxel and carboplatin were administered. In August 2018, computed tomography (CT) scans revealed a 32 × 16 mm mass in the left adnexal region, as well as a few suspicious lymph nodes with a maximum diameter of 7 × 12 mm, and a serum CA-125 level of 62.4 U/ml (normal value <35 U/ml). Debulking surgery was performed and histopathology of the left adnexal mass showed fat necrosis and benign lymph nodes. Postoperative CT scan revealed a 35 × 27 mm left adnexal mass and metastatic para-iliac lymph nodes. CA-125 levels dropped to 41.9 U/ml after three ChT cycles with Carboplatin (AUC 5), Paclitaxel 175 mg/m2, and Bevacuzimab 15 mg/m2. Despite ChT, abdominal BT revealed a 35 × 27 mm mass in the left pelvic sidewall and metastatic lymph nodes in the para-iliac region. After the third ChT cycle, the patient underwent reoperation. During the exploration, a mass under the internal iliac vein was removed. Morphology and immunohistochemistry matched high-grade ovarian serous carcinoma. In February 2020, due to paclitaxel-induced neuropathy, a ChT regimen with Gemcitabine 1000 mg/m^2^, Carboplatine (AUC 5) and Bevacuzimab 15 mg/m^2^ was preferred after surgery. In October 2020, the CA-125 level increased to 51.1 U/ml, and abdominal CT revealed a recurrent mass with a maximum diameter of 52 mm in the left adnexal region. The patient was discussed in tumor board and salvage SBRT was planned. The patient was referred to radiation oncology department for treatment with MRgRT and she was treated with 1.5 T MR-Linac (Unity® MR Linac System), Elekta AB, Stockholm, Sweden in 5 fractions to a dosage of 30 Gy to the recurrent lesion (Fig. 1). The patient was treated every other day, and there were no acute toxicity during or following MRgRT. The patients received no further treatment, and three months after MRgRT, their CA-125 level was 23.3 U/ml. After 20 months of SBRT, the patient was still disease-free without any late toxicity (Fig. 2).

### 2.2 Case 2

A 70-year-old female patient was admitted to our department with recurrent high-grade serous ovarian cancer. In June 2016, the patient had ovarian cancer and underwent total abdominal hysterectomy, left salpingo-oophorectomy, pelvic and para-aortic lymph node dissection, and appendectomy. According to histopathological findings, the patient was classified as stage IIIB and received six cycles of paclitaxel and carboplatin after surgery. In October 2018, the CA-125 level was 48.2 U/ml. The thoraco-abdominal CT scan revealed a 23 mm metastatic lesion in the right lobe posterior-superior of the segment #7 of the liver, as well as two lesions in the posterior portion of the vaginal cuff measuring 30 mm and 40 mm. The patient had an exploratory laparotomy as well as Hyperthermic Intraperitoneal Chemotherapy (HIPEC). The serum CA-125 levels returned to normal levels of 8 U/ml. Following the procedure, a ChT regimen consisting of Carboplatin (AUC 5), Paclitaxel 175 mg/m^2^, and Bevacuzimab 15 mg/m^2^ was administered up to 15 cycles.

**Fig. 3:**
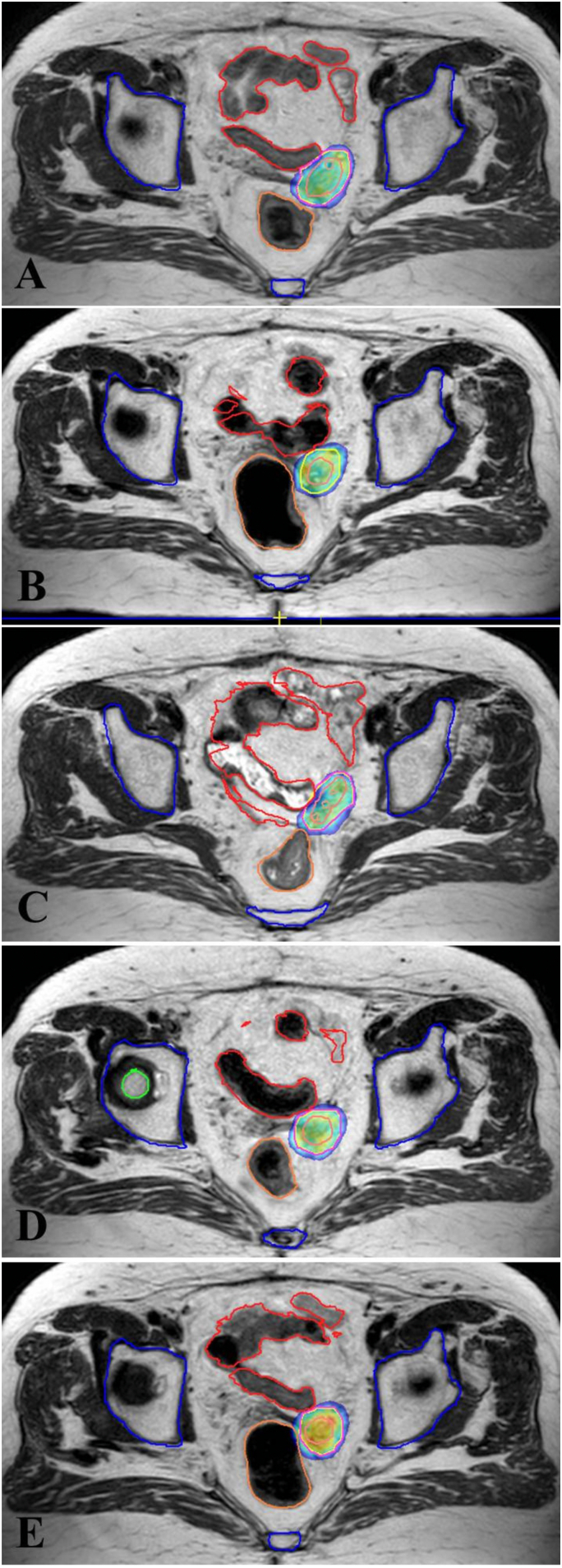
Recurrent mass located in the left part of the vaginal cuff in Case 2 treated with MR-guided stereotactic radiotherapy using online adaptive radiation therapy delivered 30 Gy to recurrent lesion in 5 fractions. 95 % isodose volumes (green area) for each fraction of the adapt-to-shape adaptive plan (A–E). Gross tumor volume (purple line), planning target volume (red line), organs at risk are bones (blue line), rectum (pink line), and bowel bag (red line). (For interpretation of the references to colour in this figure legend, the reader is referred to the web version of this article.)

**Fig. 4:**
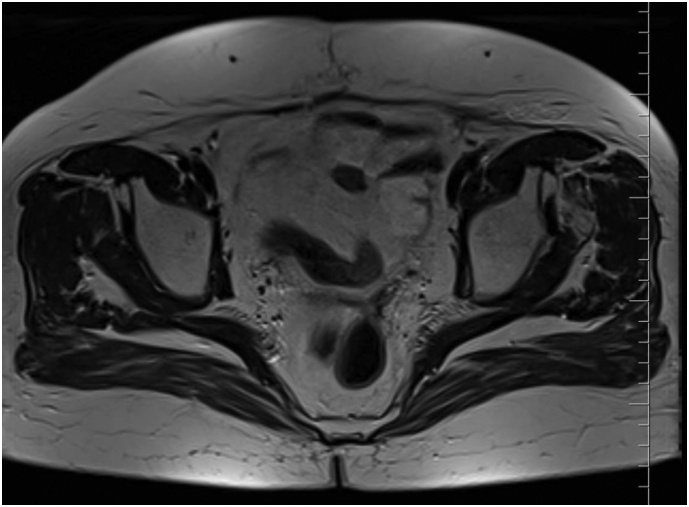
Magnetic resonance image demonstrating complete regression of lesions located at left apical part vaginal vault taken 12 months after completion of MR-guided stereotactic radiotherapy.

In April 2021 the serum CA-125 levels elevated to 29.8 U/ml, and the thoraco-abdominal CT revealed a recurrent mass located in the left part of the vaginal cuff measured as 19 × 8 mm. The patient underwent ChT with Carboplatin (AUC 5), Paclitaxel 175 mg/m2, and Bevacuzimab 15 mg/m2; however, during the second cycle, the patient developed allergic reactions and was unable to continue with the treatment. The patient was planned to have salvage SBRT after discussing the patient in tumor board. Every other day, the patient received 30 Gy of MRgRT in 5 fractions to the recurrent mass (Fig. 3). The patient did not have any acute gastrointestinal or genitourinary toxicity during or following MRgRT, and Ca-125 levels dropped to 9.1 U/ml. After 12 months of MRI-g-SBRT, the patient is still disease-free, and with no late toxicity (Fig. 4).

## 3 Discussion

High-grade serous carcinoma is the most common histological subtype of EOS, and it typically presents at an advanced stage, primarily disseminating throughout the peritoneal cavity prior to the development of any symptoms. Because high grade serous carcinoma is known to be sensitive to platinum-based ChT, the role of adjuvant whole abdominal irradiation (WAI) in sterilizing micro-metastatic disease has declined. Moreover, the main disadvantage of WAI was its dose-limiting toxicities, which were mostly acute hematologic and late gastrointestinal in nature [5].

The most common pattern of treatment failure in EOC patients is abdomino-pelvic recurrence. Failures after first-line therapy have a poor prognosis, particularly for those who relapsed <6 months after treatment completion, indicating platinum-resistant disease. Currently, RT is used in selected patients with EOC for limited loco-regional recurrences, oligometastatic or oligoprogressive disease, and pain relief. The majority of the studies included both nodal and extra-nodal recurrences and used tumor directed RT utilizing more conformal RT techniques, with a reported in-field disease control of approximately 70 % at 5 years [8], [9]. Non-serous histology, platinum sensitivity, biologically effective radiation doses (BED) higher than 35 Gy, and nodal recurrences were identified as factors associated with a lower relapse risk [4], [9], [10]. We utilized salvage irradiation in both cases due to the unfavorable local control factors of serous histology and extra-nodal relapse. Moreover, despite undergoing multiple cytoreductive surgeries, the patients' diseases were platinum-sensitive, and the location of the relapse tumor made surgery difficult.

Because of technological advancements, RT techniques have evolved significantly over the last few decades. Stereotactic body radiotherapy (SBRT) has been a game changer in recent decades, with complex dose calculation algorithms and image guided RT allowing for increasingly precise target coverage while sparing nearby healthy tissues. Radiation oncologists are currently witnessing the implementation of the most recent technological revolution: the combination of MRI and a linear accelerator, known as MR-Linac. MRgRT is a novel and rapidly evolving technology that has the potential to improve the risk-benefit ratio for cancer patients [11]. Direct visualization of the tumor and surrounding healthy tissues during treatment enables extremely precise treatment of even mobile targets. Each treatment can be tailored to the patient's changing anatomy, potentially reducing the risk of radiation-related side effects while increasing the tumor-targeted dose. High doses of radiation have been shown to be required for the treatment of EOC in a curative setting. The majority of EOC patients, however, frequently present with recurrences in the abdomino-pelvic region. It was difficult to prescribe higher radiation doses for the eradication of any lesion without inducing high rates of toxicity. In our cases, the target volumes are located close to the intestines. To deliver higher doses to target volumes while sparing surrounding tissues, organ motion must be monitored during treatment, which MR-Linac enables. In a phase I trial, Henke et al. [12] evaluated the treatment outcomes of 10 patients with recurrent OC treated with MRgRT utilizing the SMART technique and a dose of 35 Gy delivered over the course of five days. At three months, the local control rate was 94 %, the median progression-free survival was 10.9 months, and only one patient experienced acute Grade 3 treatment-related toxicity (duodenal ulcer). Both of our cases tolerated the treatment well, with no acute or late serious toxicity, and the patients were disease-free after 12 and 20 months of MRgRT, respectively.

## 4 Conclusion

Our findings suggested that MRgRT may be an effective treatment option for locoregionally recurrent EOC that does not cause severe radiation-related toxicity. Due to the technical difficulties involved in delivering high radiation doses in a safe manner, RT is rarely used in patients with a highly selected EOC. Nonetheless, with the implementation of high field 1.5-T MR with RT devices, it is simple to identify tumors in soft tissues and to monitor organ motion throughout the treatment session. This innovative technique enables the delivery of hypofractionated RT with tight safety margins to reduce radiation doses to nearby normal tissues. The role of RT in patients with persistent or recurrent EOC should be reconsidered, regardless of initial resistance or gradual development of intolerance to standard chemotherapy regimens. However, additional research is required to determine the impact of this new method on EOC patient outcomes.

## Provenance and peer review

Not commissioned, externally peer-reviewed.

## Consent

Written informed consent was obtained from the patient for publication of this case report and accompanying images. A copy of the written consent is available for review by the Editor-in-Chief of this journal on request.

## Guarantor

Cem Onal, MD

## Ethical approval

This case report does not require any ethical approval.

## Funding

This research did not receive any specific grant from funding agencies in the public, commercial, or not-for-profit sectors.

## CRediT authorship contribution statement

Guler Yavas MD - Study concept, writing the paper, final draft.

Ulku Esra Kuscu, MD - Data collection, review of literature, final draft.

Cagdas Yavas, MD - Data curation, resources, investigation, visualization.

Cem Onal, MD - Conceptualization, formal analysis, investigation, writing - review & editing, supervision.

Ali Ayhan, MD - Data collection, review of literature, supervision.

## Declaration of competing interest

There were no conflicts of interest.
